# Inner Nuclear Membrane Asi Ubiquitin Ligase Catalytic Subunits Asi1p and Asi3p, but not Asi2p, confer resistance to aminoglycoside hygromycin B in *Saccharomyces cerevisiae*

**DOI:** 10.17912/micropub.biology.000403

**Published:** 2021-06-01

**Authors:** Kelsey A Woodruff, Kyle A Richards, Melissa D Evans, Abigail R Scott, Brian M Voas, Courtney Broshar Irelan, James B Olesen, Philip J Smaldino, Eric M Rubenstein

**Affiliations:** 1 Ball State University

## Abstract

The heterotrimeric Asi ubiquitin ligase (encoded by *ASI1*, *ASI2*, and *ASI3*) mediates protein degradation in the inner nuclear membrane in *Saccharomyces cerevisiae*. Asi1p and Asi3p possess catalytic domains, while Asi2p functions as an adaptor for a subset of Asi substrates. We hypothesized the Asi complex is an important mediator of protein quality control, and we predicted that Asi would be required for optimal growth in conditions associated with elevated abundance of aberrant proteins. Loss of Asi1p or Asi3p, but not Asi2p, sensitized yeast to hygromycin B, which promotes translational infidelity by distorting the ribosome A site. Surprisingly, loss of quality control ubiquitin ligase Hul5p did not sensitize yeast to hygromycin B. Our results are consistent with a prominent role for an Asi subcomplex that includes Asi1p and Asi3p (but not Asi2p) in protein quality control.

**Figure 1. ASI1 and ASI3 confer resistance to hygromycin B:**
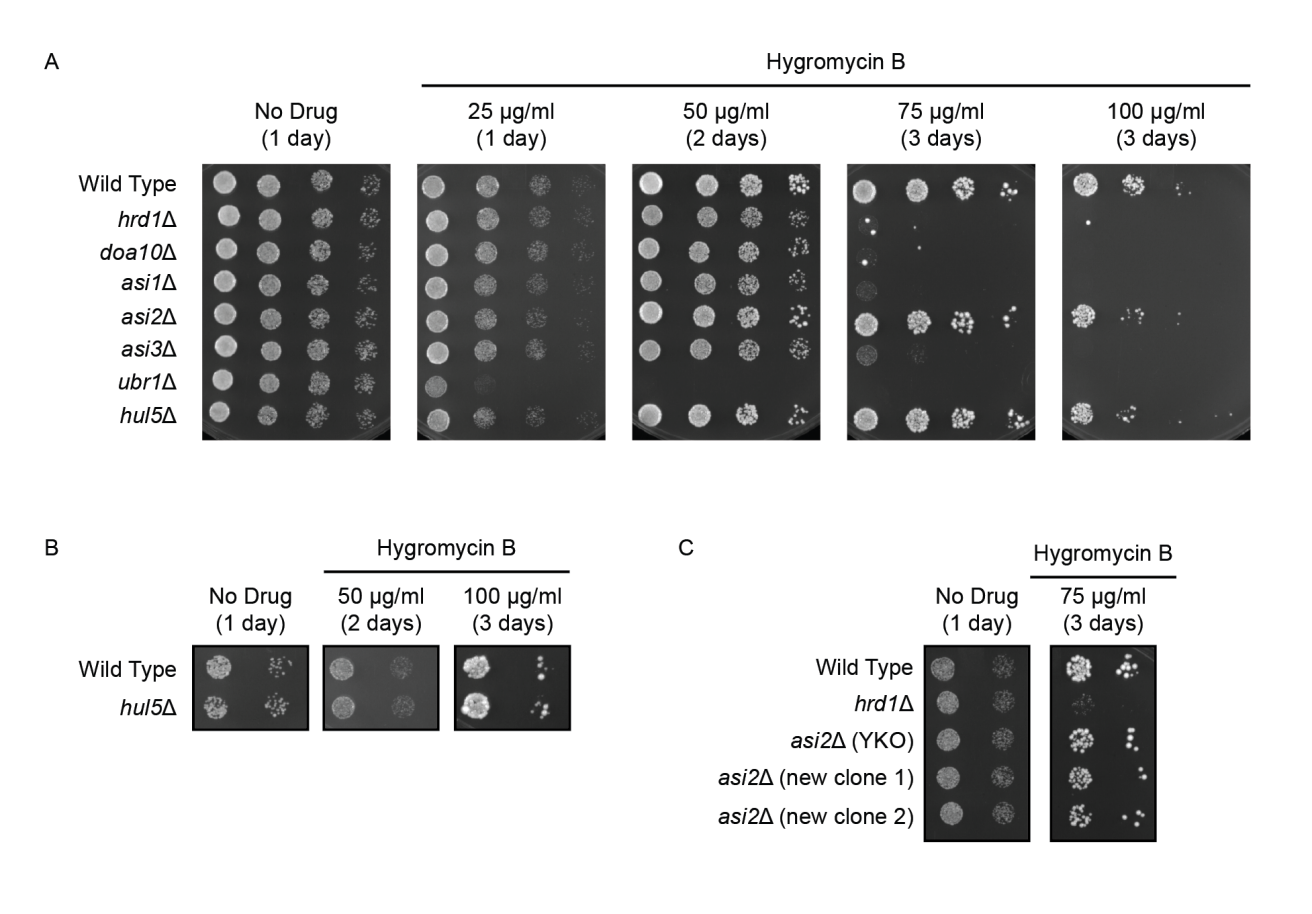
**(A-C)** Sixfold serial dilutions of yeast of the indicated genotypes were spotted onto agar plates containing rich growth medium (No Drug) or indicated concentrations of hygromycin B. Plates were incubated at 30°C and imaged after 1-3 days. Experiments were performed in triplicate. **(C)** “*asi2Δ* (YKO)” is VJY852 and was obtained from the Yeast Knockout Collection (Tong *et al.*, 2001). “*asi2Δ* (new clone 1)” and “*asi2Δ* (new clone 2)” are VJY969 and VJY970, respectively, and were generated for this study.

## Description

Organelle proteome maintenance is essential for eukaryotic life. Several dedicated proteolytic systems promote organelle-specific turnover of misfolded or excess proteins. Inner nuclear membrane (INM) proteins are targeted for proteasomal destruction via INM-associated degradation (INMAD). At least three ubiquitin ligases mediate INMAD in the budding yeast *Saccharomyces cerevisiae*. These include the Asi complex, Doa10p, and the anaphase promoting complex (Deng and Hochstrasser, 2006; Foresti *et al.*, 2014; Khmelinskii *et al.*, 2014; Koch *et al.*, 2019). Asi is composed of three subunits, Asi1p, Asi2p, and Asi3p (Foresti *et al.*, 2014; Khmelinskii *et al.*, 2014). Asi1p and Asi3p possess catalytic Really Interesting New Gene (RING) domains, while Asi2p serves as an adaptor for a subset of Asi substrates (Foresti *et al.*, 2014; Khmelinskii *et al.*, 2014; Natarajan *et al.*, 2020).

Asi contributes to protein quantity control (e.g. degradation of orphan subunits of oligosaccharyl transferase and glycosylphosphatidylinositol transamidase complexes (Natarajan *et al.*, 2020)) and localization control (e.g. degradation of mislocalized ergosterol synthetic enzyme Erg11p (Buchanan *et al.*, 2019; Natarajan *et al.*, 2020) and of transcription factors Stp1p and Stp2p when they inappropriately enter the nucleus (Natarajan *et al.*, 2020; Omnus *et al.*, 2011). The Asi complex and the endoplasmic reticulum-localized ubiquitin ligase Hrd1p redundantly promote the degradation of mutated, hypofunctional translocon subunit sec61-2p (Foresti *et al.*, 2014; Trueman *et al.*, 2011), suggesting Asi may also mediate protein quality control (PQC) of misfolded polypeptides. Only Asi1p and Asi3p (but not Asi2p) promote sec61-2p degradation (Foresti *et al.*, 2014) and mitigate toxicity caused by sec61-2p expression (Flagg *et al.*, 2021), raising the possibility that PQC function of Asi is mediated by Asi1p and Asi3p alone, or in conjunction with unidentified substrate specificity factors.

The aminoglycoside hygromycin B produced by the bacterium *Streptomyces hygroscopicus* reduces translational fidelity by distorting the ribosome A site, resulting in inaccurately synthesized protein molecules (Brodersen *et al.*, 2000; Ganoza and Kiel, 2001). We previously demonstrated that loss of ER and nuclear PQC ubiquitin ligases Hrd1p, Doa10p, and Ubr1p sensitizes cells to hygromycin B (Crowder *et al.*, 2015; Niekamp *et al.*, 2019; Runnebohm *et al.*, 2020). The extent of Asi’s contribution to PQC relative to these enzymes remains unknown.

We hypothesized that Asi is an important mediator of PQC. We predicted that the Asi complex would be required for resistance to conditions expected to increase the abundance of aberrant proteins. To test this, we cultured wild type yeast, yeast lacking genes encoding each subunit of the Asi complex, and a panel of PQC mutant yeast strains in the absence and presence of increasing concentrations of hygromycin B (Figure 1A). Consistent with previous results, loss of *HRD1* or *DOA10* sensitized cells to 75 μg/ml hygromycin B, and yeast deleted for *UBR1* exhibited sensitivity at concentrations as low as 25 μg/ml. By contrast, deletion in two different genetic backgrounds of the gene encoding PQC ubiquitin ligase Hul5p (Fang *et al.*, 2011; Runnebohm *et al.*, 2020; Sitron and Brandman, 2019) did not sensitize cells to hygromycin B at the concentrations evaluated (Figure 1A, 1B).

Loss of *ASI1* and *ASI3* sensitized cells to 75 μg/ml hygromycin B to a similar extent as loss of *DOA10* or *HRD1* (Figure 1A). Intriguingly, loss of *ASI2* in multiple independently generated yeast strains did not confer a similar growth disadvantage under these conditions (Figure 1A, 1C). Deletions of *ASI* genes and *HUL5* were validated by PCR. Taken together, our results indicate Asi1p and Asi3p, but not Asi2p, are required for optimal growth in the presence of a compound expected to generate increased numbers of PQC substrates.

The finding that loss of Hul5p does not enhance sensitivity to hygromycin B was surprising, given multiple characterized functions of Hul5p in PQC. Among other roles, Hul5p promotes degradation of substrates that have escaped detection by the ribosome quality control ubiquitin ligase Ltn1p (Sitron and Brandman, 2019) and promotes turnover of misfolded proteins following heat shock (Fang *et al.*, 2011). Loss of Ltn1p sensitizes cells to hygromycin B (Bengtson and Joazeiro, 2010; Crowder *et al.*, 2015). We speculate that a requirement for Hul5p in hygromycin B resistance may become apparent during conditions characterized by elevated cellular dependence on Hul5p, such as compromised Ltn1p function or heat shock.

Multiple lines of evidence suggest that a subcomplex of the Asi ubiquitin ligase including Asi1p and Asi3p (but not Asi2p) mediates PQC degradation of misfolded proteins, potentially in complex with unidentified substrate adaptors. First, as demonstrated here, deletion of *ASI1* or *ASI3*, but not of *ASI2*, sensitizes cells to conditions expected to increase the abundance of aberrant, mistranslated proteins to an extent similar to that observed following loss of other characterized PQC genes (we note it remains possible that *ASI2* is required for optimal growth under different forms of proteotoxic stress, such as elevated temperature). Second, while Asi1p, Asi2p, and Asi3p collaborate to mediate degradation of a host of mislocalized proteins, only Asi1p and Asi3p promote degradation of mutated translocon component sec61-2p (Foresti *et al.*, 2014). Finally, simultaneous deletion of genes encoding Hrd1p, Ire1p (a component of the yeast unfolded protein response), and either Asi1p or Asi3p causes markedly slower growth than concurrent knockout of *HRD1*, *IRE1*, and *ASI2* (Foresti *et al.*, 2014).

Asi2p function is also dispensable for degradation of some Asi1/3p substrates that do not possess features rendering them predicted PQC substrates (Khmelinskii *et al.*, 2014). Such substrates may expose degradation signals (e.g. when other complex subunits are present in substoichiometric abundance) resembling those of quality control substrates, co-opting a PQC enzyme for regulatory purposes. The precise nature of degradation signal(s) recognized by Asi remains to be resolved.

## Methods

***ASI2* gene replacement.** To generate VJY969 and VJY970, *ASI2* was replaced with *kanMX4* via homologous recombination. An 1807-bp *asi2Δ*::*kanMX4* cassette was PCR-amplified from VJY852 (Yeast Knockout Collection *asi2Δ*::*kanMX4* strain (Tong *et al.*, 2001)) using primers VJR472 (5’ GACACCGAATCAAACGCATA 3’) and VJR473 (5’ GGAAAGCTTGCAAACAGCTC 3’) and introduced into naïve wild type VJY476 (alias BY4741 (Tong *et al.*, 2001)) by lithium acetate transformation (Guthrie and Fink, 2004). G418-resistant clones were validated by PCR genotyping at the 5’ and 3’ recombination junctions.

**Yeast growth assay.** Yeast growth analysis was performed as described (Watts *et al.*, 2015). Four μl of sixfold serial dilutions were pipetted onto yeast extract-peptone-dextrose medium (Guthrie and Fink, 2004) in the absence or presence of increasing concentrations of hygromycin B (Gibco). Plates were incubated at 30°C and imaged at the indicated times.

## Reagents

**Table:** 

**Name**	**Genotype**	**Figure**	**Reference**
VJY60 (alias W303)	*MATa ade2-1 ura3-1 his3-11 trp1-1 leu2-3,112 can1-100*	1B	(Thomas and Rothstein, 1989)
VJY85	*MATa ade2-1 ura3-1 his3-11 trp1-1 leu2-3,112 can1-100 hul5Δ::LEU2*	1B	(Wang *et al.*, 1999)
VJY360	*MATa his3Δ1 leu2Δ0 met15Δ0 ura3Δ0 asi1Δ::kanMX4*	1A	(Tong *et al.*, 2001)
VJY469	*MATa his3Δ1 leu2Δ0 met15Δ0 ura3Δ0 ubr1Δ::kanMX4*	1A	(Tong *et al.*, 2001)
VJY476 (alias BY4741)	*MATa his3Δ1 leu2Δ0 met15Δ0 ura3Δ0*	1A, 1C	(Tong *et al.*, 2001)
VJY511	*MATa his3Δ1 leu2Δ0 met15Δ0 ura3Δ0 hrd1Δ::kanMX4*	1A, 1C	(Tong *et al.*, 2001)
VJY662	*MATa his3Δ1 leu2Δ0 met15Δ0 ura3Δ0 hul5Δ::kanMX4*	1A	(Tong *et al.*, 2001)
VJY667	*MATa his3Δ1 leu2Δ0 met15Δ0 ura3Δ0 doa10Δ::kanMX4*	1A	(Tong *et al.*, 2001)
VJY696	*MATa his3Δ1 leu2Δ0 met15Δ0 ura3Δ0 asi3Δ::kanMX4*	1A	(Tong *et al.*, 2001)
VJY852	*MATa his3Δ1 leu2Δ0 met15Δ0 ura3Δ0 asi2Δ::kanMX4*	1A, 1C	(Tong *et al.*, 2001)
VJY969	*MATa his3Δ1 leu2Δ0 met15Δ0 ura3Δ0 asi2Δ::kanMX4*	1C	This study
VJY970	*MATa his3Δ1 leu2Δ0 met15Δ0 ura3Δ0 asi2Δ::kanMX4*	1C	This study
